# Evaluation of the Hypoglycemic Properties of *Anacardium humile* Aqueous Extract

**DOI:** 10.1155/2013/191080

**Published:** 2013-05-02

**Authors:** Márcio A. Urzêda, Silvana Marcussi, Luciana L. Silva Pereira, Suzelei C. França, Ana Maria S. Pereira, Paulo S. Pereira, Saulo L. da Silva, César L. S. Guimarães, Leonardo A. Calderon, Rodrigo G. Stábeli, Andreimar M. Soares, Lucélio B. Couto

**Affiliations:** ^1^Unidade de Biotecnologia, Universidade de Ribeirão Preto, UNAERP, Ribeirão Preto, SP, Brazil; ^2^Departamento de Química, Universidade Federal de Lavras, UFLA, Lavras, MG, Brazil; ^3^Universidade Federal de São João Del Rei, UFSJ, Campus Alto Paraopeba, Ouro Branco, MG, Brazil; ^4^Centro de Estudos de Biomoléculas Aplicadas à Saúde, CEBio, Fundação Oswaldo Cruz, FIOCRUZ Rondônia e Departamento de Medicina, Universidade Federal de Rondônia, UNIR, Porto Velho, RO, Brazil; ^5^Instituto Brasileiro do Meio Ambiente e dos Recursos Naturais Renováveis, IBAMA, Porto Velho, RO, Brazil

## Abstract

The antihyperglycemic effects of several plant extracts and herbal formulations which are used as antidiabetic formulations have been described and confirmed to date. The main objective of this work was to evaluate the hypoglycemic activity of the aqueous extract of *Anacardium humile*. Although the treatment of diabetic animals with *A. humile* did not alter body weight significantly, a reduction of the other evaluated parameters was observed. Animals treated with *A. humile* did not show variation of insulin levels, possibly triggered by a mechanism of blood glucose reduction. Levels of ALT (alanine aminotransferase) decreased in treated animals, suggesting a protective effect on liver. Levels of cholesterol were also reduced, indicating the efficacy of the extract in reestablishing the balance of nutrients. Moreover, a kidney protection may have been achieved due to the partial reestablishment of blood glucose homeostasis, while no nephrotoxicity could be detected for *A. humile*. The obtained results demonstrate the effectiveness of *A. humile* extracts in the treatment of alloxan-induced diabetic rats. Therefore, *A. humile* aqueous extract, popularly known and used by diabetic patients, induced an improvement in the biochemical parameters evaluated during and following treatment of diabetic rats. Thus, a better characterization of the medicinal potential of this plant will be able to provide a better understanding of its mechanisms of action in these pathological processes.

## 1. Introduction

Plants have been used for years as a source of traditional medicine to treat various diseases. Many of these medicinal plants are also excellent sources for phytochemicals, many of which contain potent antioxidant activities [1]. The study of medicinal plants as therapeutic agents is a huge approach regarding health problems of indigenous communities and third world countries. In Brazil, the influence of indigenous, African, and European cultures regarding the use of these plants is widely reported, evidencing the knowledge and use of these plants by indigenous communities until now [2]. Several medicinal plants have been used in traditional medicine for the treatment of diabetic patients in different ethnic societies of Africa, Asia, and South America [3]. The antihyperglycemic effects of several plant extracts and herbal formulations which are used as antidiabetic formulations have been confirmed [4].

Although other researchers had already noticed the presence of sugar in the urine of diabetics, this was demonstrated by Dobson only in 1755 [4]. *Diabetes mellitus* characterizes the most common endocrine disease worldwide, reaching about 173 million people, and it is estimated that this number will more than double over the next 25 years. According to Funke and Melzig [5], this disorder is characterized by chronic hyperglycemia with disturbances of metabolism resulting from lack of insulin secretion and insufficient cellular production of insulin.

Diabetes is now the fourth or fifth leading cause of death in most developed countries, and with people suffering from the disease worldwide, its incidence is approaching epidemic proportions [6].

Diabetes is significantly (30% to 50%) related to diseases as ischemic cardiopathy, heart failure, cerebral vascular accident and systemic arterial hypertension [7]. The incidence (25%) of cardiovascular diseases (acute infarct) and mortality are increased in diabetic patients [8]. *Diabetes mellitus* is an apprehensive disease due to its diffused characteristics that, progressively, induce various pathologies with severe consequences.

Diabetes therapies are associated with the control of the signs and symptoms of the pathologies without, however, promoting the disease cure. The currently available treatment options for hyperglycemia, apart from lifestyle changes and weight reduction, are oral hypoglycemic agents (OHAs) with various modes of action, and different types of insulin [9]. Many classes of medicines are used for the treatment of diabetes, but all of them show adverse effects. Several approaches are described to reduce the hyperglycemia, such as treatment by sulfonylureas, which stimulates pancreatic islet cells to secrete insulin and metformin, reducing the hepatic glucose production [10]. All these therapies have limited efficacy and various side effects, and thus searching for new classes of compounds is essential to overcome these problems. Thus, it has increased the demand for low-cost alternatives to aid in controlling blood glucose levels, to prevent or delay the onset of complications related to diabetes, based on pharmaceutical ethnobotany [11].

Many plants have been reported to present hypoglycemic activity in the last years. In Brazil, the popular use of medicinal plants to control diabetes is very common. *Anacardium humile* St.-Hil., a species from the Anacardiaceae family, native to the Brazilian cerrado, has been popularly acclaimed against diabetes. This plant is a shrub (30 cm tall), with very long roots, small flowers, a greenish calyx, and red petals with stripes [12]. *Anacardium* species are known for their anti-inflammatory and astringent effects, activity against cancer cells, and their beneficial effects on gastrointestinal ulcers [12–14]. The aim of this work was to evaluate the hypoglycemic properties of the aqueous extract of *A. humile* on alloxan-induced diabetic rats.

## 2. Materials and Methods

### 2.1. Plant Material

Phloem of *A. humile* was collected in the Brazilian cerrado, region of Araxá, Minas Gerais, Brazil, 19^o^43′04,0′′, W 46^o^53′54,0′′ and 949 m of altitude WGS84 datum, coordinates measured by Garmin, model Legend global positioning system. Plant material was dried in a circulating air oven at 50°C for 24 h and then ground in a knife mill until total pulverization. The stems from *A. humile* were washed and dried to constant weight in a forced air circulating drier at 40 ± 5°C. Dried stems were ground and stored at *∼*4°C until use. To make an aqueous extract, the ground dried stems (100 g) were placed in a beaker containing deionized water (1 : 5 w/v) and held in a water bath at 60°C for 60 min and then hot-filtered through a gauze funnel. After cooling, the solution was frozen, lyophilized, and stored at −20°C, thereby enabling the weighing and dilution in deionized water. Before use, the freeze-dried aqueous extracts were weighed, dissolved in saline, and centrifuged at 10,000 ×g for 10 min, and the supernatants (stock solutions at 250 *μ*g/*μ*L) were stored at −20°C.

### 2.2. Animals and Diets

Male Wistar rats (*Rattus norvegicus,* 8 weeks old; 200 ± 20 g) were grown in the Animal Facilities of the University of Ribeirão Preto-UNAERP. The animals were housed in polypropylene cages at room temperature (20–25°C) under 12 h light-dark photoperiod. During the experiment, the rats received *ad libitum* water and standard pellet diet (Nuvilab) composed of (wet weight) 66% carbohydrates, 16% proteins, 8% fat, and 10% micronutrients-vitamins-mineral salts for six days. After diabetes induction, the animals were isolated in metabolic cages with daily replacement of food (45 g) and water (200 mL) at 8:00 AM. The experiments described were approved by Institutional Committee for Ethics in Animal Experimentation and were done in accordance with the guidelines of the Brazilian College for Animal Experimentation (COBEA).

### 2.3. Induction of the Experimental Diabetes

Prior to diabetes induction, the animals were kept fasting for 12 hours. The inducing agent used was alloxan, dissolved in saline solution and injected intraperitoneally (100 mg/kg) within five minutes after dissolution [15, 16]. Considering that glucose administration protects the animals against the diabetogenic action of alloxan [17], the animals were kept fasting in order to avoid excessive accumulation of feeding glucose, which would antagonize the alloxan effects [18]. The volume of alloxan injection was calculated according to the animal body weight, observing the maximum volume limit of 0.5 mL, and the control group received equal volume of saline solution. Two hours after diabetes induction, the animals received *ad libitum* diet. Forty-eight hours after alloxan administration, diabetes was verified by blood glucose (manual glucometer Advantage II) and animals that showed glucose concentration higher than 250 mg/dL were selected. 

After the isolation period, the animals (*N* = 18) were distributed in three groups of 6 rats each: Group (1) non-diabetic control rats (C) and group (2) untreated diabetic rats (D) and Group (3) diabetic rats treated with the aqueous extract of *A. humile* (T). Clinical parameters as the ingestion of water and food, excretion of urine, body weight and the biochemical parameters as glucose, urea, creatinine and urinary proteins were evaluated on day 2. The glycemic parameter was checked weekly using a manual glucometer (Advantage II). Total cholesterol, alanine aminotransferase (ALT) and insulin were measured on the 28th day.

### 2.4. Administration of the *A. humile* Aqueous Extract

After 14 days, blood glucose was measured to exclude animals with possible spontaneous reversions of *diabetes mellitus* induced earlier and the animals were divided into three groups for the experiment. Rats were treated by gavage, using stainless steel tube and syringes, twice a day at 8:00 am and 8:00 pm during 28 days, by the same handler, respecting the maximum volume of 1 mL per animal. The animals of the T group received the dose of 175 mg/kg of aqueous extract of *A. humile* in variable volumes according to the animal weight. Diabetic rats of the D group as well as the rats of the C group received only saline solution.

### 2.5. Sampling and Tests

Urine was collected in plastic flasks every day, discarding the samples of the first 24 hours. The urinary volumes of the samples were measured, homogenized, placed in 10 mL tubes, and centrifuged at 11,200 ×g for 5 minutes. Analyses to determine the levels of glucose, urea, creatinine and protein were accomplished one hour after centrifugation. Glycemia tests were carried out using a glucometer. For the determination of plasma glucose levels, the tip of the rat tail was cut and the vein blood was collected weekly from all groups (between 08:30 and 09:00 h). At the end of the experiment (28 days), the animals were anesthetized (40 mg/kg, i.p.) with sodium thiopental (Tiopental). When rats were under deep anesthesia, a ventral midline incision was made, and 3 mL of blood was collected from the abdominal aorta and gently placed in Vacutainer tubes, containing a coagulation activator to avoid hemolysis. After coagulation, the material was centrifuged at 1,550 ×g for 5 minutes to separate the serum from the fibrin mesh. Biochemical analyses were performed within one hour after sampling.

### 2.6. Analytical Procedures

The laboratorial analyses were carried out at the Laboratory of Clinical Analysis of the University of Ribeirão Preto (UNAERP). Glucose levels in serum and urine were measured using the hexokinase method; urea in urine and total cholesterol were determined by the enzymatic method; urinary proteins were evaluated by a colorimetric method. All analyses were performed using Labtest kit (Bayer). The quantitative determination of ALT was performed employing the method optimized by the International Federation of Clinical Chemistry (IFCC) using the Biosystems test kit. Insulin was determined by the chemiluminescence method using the Kit Ultrasensitive insulin 33410-Beckman.

### 2.7. Statistical Analysis

All collected data represent the average ± standard deviations (S.D.). Comparisons between the different groups were carried out by analysis of variance followed by the Tukey test. Differences were considered statistically significant when *P* < 0.05.

## 3. Results and Discussion

**Figure 1:**
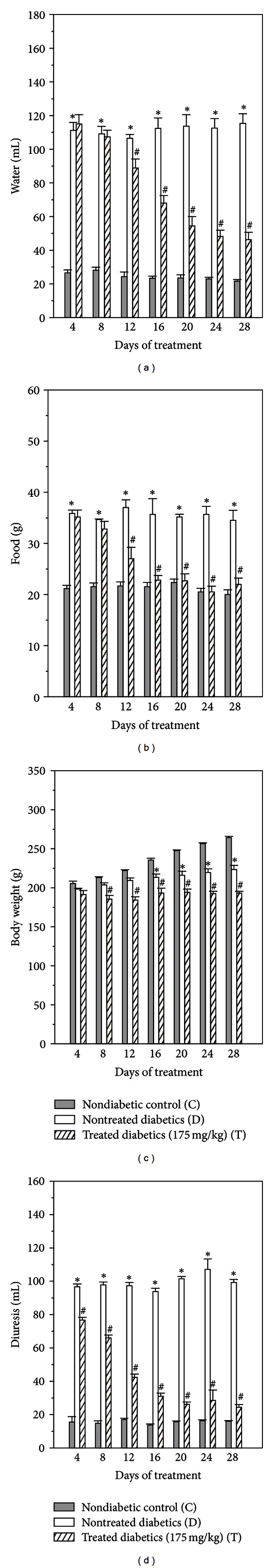
Evaluation of clinical parameters: average ± SD. (a) Water ingestion; (b) food ingestion; (c) body weight; (d) diuresis (mL). *P < 0.05 between groups C and D; ^#^ *P* > 0.05 between groups D and T.

**Figure 2:**
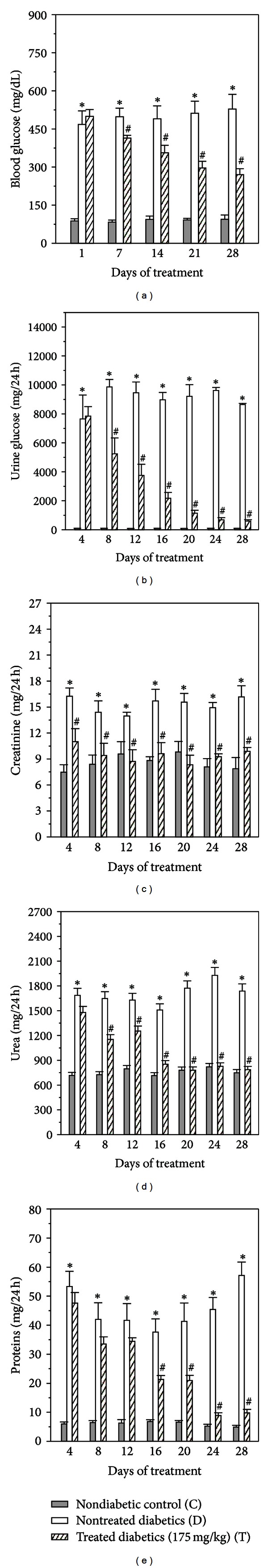
Evaluation of biochemical parameters: average ± SD. (a) Blood glucose; (b) urine glucose; (c) urinary creatinine; (d) urinary urea; (e) urinary proteins. *P < 0.05 between groups C and D; ^#^ *P* > 0.05 between groups D and T.

Alloxan was able to produce diabetes in rats, with the development of clinical and laboratory definition, including elevation of water intake (Figure 1(a)), and urine output (Figure 1(d)), blood glucose levels above 400 mg/dL (Figure 2(a)) and urinary glucose greater than 7000 mg in 24 h (Figure 2(b)).

The present study demonstrated that the acute hyperglycemia induced by alloxan in rats was reduced by the administration of *A. humile* aqueous extract (175 mg/kg) by gavage twice a day during 28 days (Figures 2(a) and 2(b)). A similar effect was reported by Kamtchouing et al. [19] using the same dosage of *A. occidentale* aqueous extract. The effects of *A. humile* on blood glucose levels were very relevant, since blood collection was not performed during fasting, but with animals being fed *ad libitum*. 

Figueroa-Valverde et al. [20] attributed the hypoglycemic effect of *Cnidoscolus chayamansa* to the presence of amentoflavone. Additionally, a study accomplished by Ferreira [21] with the extract and fractions obtained from *A. humile* showed that the majority of substances present in this species of plant are flavonoids derived from quercetin, an amentoflavone biflavonoid, derived from gallic acid and tannins. Therefore, flavonoids like amentoflavone may be related to the hypoglycemic effect observed in this study.

The treatment of diabetes with *A. humile* promoted a reduction of 32% in food intake (Figure 1(b)), comparing rats at the first and last days of treatments when the animals presented almost normal glucose levels, probably as a consequence of the consumption of available glucose in the blood [22]. Also, the synthesis of proteins and lipids may have contributed to the decrease of food ingestion, considering that the high levels of creatinine, urea, and urinary proteins found for the diabetic control group indicate a lower protein synthesis and a higher protein excretion in these animals compared to the treated group (Figures 2(c), 2(d), and 2(e)). The accentuated and rapid loss of weight is one of the first reasons that lead an individual to search for medical care [23]. In our study, significant alterations in body weight were observed in treated animals. However, an enhanced weight gain (32%) was observed (Figure 1(c)) in control rats, which may be related to the effective production of insulin in response to nourishment. According to Cha et al. [24], the significance of insulin for the physical development functioning is associated with the growth hormone (GH). Diabetic animals treated with *A. humile* presented a low gain of weight, suggesting a possible secondary effect of this plant in reducing or inhibiting growth. It is possible that *A. humile* causes an inhibition of *α*-amylase secretion into the intestine, improving the metabolism of glucose and triglyceride, as observed by Chen et al. [25] using *Rhizoma polygonati odorati* extracts. In this work lower gain of weight was observed in the diabetic animals treated with the aqueous extract, which presented a reduction of 2.37% in weight gain. This hypothesis may be confirmed by the lack of alterations in the insulin synthesis, which also acts as a growth hormone [26]; however, additional studies are necessary. 

In the present work it was verified that the administration of *A. humile* significantly reduced water ingestion from the twelfth day to the end of the treatment (Figure 1(a)). It was observed that the ingestion of water by control rats did not achieve significant variation, probably because blood glucose did not return to normal levels. At the end of the experiments, diabetic treated rats reduced by 61.23% the ingestion of water compared to the fourth day of treatment. The effect of *A. humile* on diuresis seems to be related to its effectiveness in reducing blood glucose. However, the decrease in glucose in the treated group causes an improvement in the characteristic symptoms of hyperglycemia, polyphagia among them, resulting in a lower food intake.

 Treated rats showed decreased diuresis with a reduction of 68.33% being observed in the urinary volume at the end of the treatment (Figure 1(d)). The polydipsia present in diabetic animals is due to blood hyperosmolarity, due to the high levels of circulating glucose, which causes the movement of water of the extracellular to the intracellular space, in order to maintain osmotic equilibrium. The intracellular dehydration is sensed by osmoreceptors in the brain, triggering intense thirst. The improvement in clinical status due to lower blood glucose in the treated group may be evidenced by the significant decrease in polyuria and polydipsia at the end of the experiment.

The administration of *A. humile* was showed to significantly reduce the urine proteins from the 16th day of treatment in (Figure 2(e)), suggesting some kind of protective effect of this extract on the nephropathy induced by diabetes. However, simple microalbuminuria laboratory tests may not be effective in predicting a diabetic nephropathy, which makes additional biochemical analysis necessary, as for urine creatinine levels [27].

Dosing urine proteins is of great help for evaluating diabetes treatment, since it may indicate diabetic nephropathy, which may be preceded by a period of increased urinary albumin excretion (UAE; 20–300 mg/24 h), defined as microalbuminuria [28]. Urine protein dosage is also effective in evaluating hypoglycemic activity of plant extracts and in determining the risk of renal lesions by plant active principles [29]. Grindley et al. [30] reported an increase of proteinuria (nephropathy) after administration of Yam (*Dioscorea cayenensis*), considered a hypoglycemiant plant. 

Dornas et al. [31] reported that nephrotoxicity induced by gentamicin can be attenuated by gallic acid, one of the major components among the phenolic compounds present in the extract of *A. humile*. According to these authors, the phenolic compounds participate in increasing the antioxidant function, and due to their action as metal chelators and enzyme modulators, they may attenuate renal damage caused by oxidative changes. In some cases this possible protection of renal function is consistent with the increase in urine creatinine levels; however, it was not observed in the present study (Figure 2(c)). Creatinine produced by creatine is a chemical waste molecule that is, generated from muscle metabolism and excreted entirely by the kidneys. Bwititi et al. [32] showed an absence of nephrotoxicity in diabetic rats and confirmed a renal protection of *Opuntia megacantha* treated animals, which presented decreases in glomerular filtration rate. In spite of the revealed protection, additional analysis, as kidney histopathology, is necessary to discard the inexistence of any renal lesions [33]. 

Results showed that the administration of *A. humile* aqueous extract decreased (41.75%) the excretion of urine urea along the treatment (Figure 2(d)). Moreover, it was observed that, after the twelfth day of treatment, the urine urea levels were similar for both the control and treated rats, emphasizing the effectiveness of the extract in reducing rates of urea excretion. According to Pepato et al. [34], the decrease of blood glucose causes a reduction of urea renal excretion. Our data suggest a partial glucose homeostasis by the administration of *A. humile* aqueous extract.

High levels of glucose are a major symptom of diabetes and the detection of glucose in the urine is relevant in selecting the treatment against this pathology, once there is a direct correlation between glucose concentration in the urine and in the blood [35]. The administration of *A. humile* extract in diabetic rats significantly reduced the levels of glucose excretion in the urine compared with results obtained for untreated rats (Figure 2(b)). Glucose in urine of T group animals was similar to the animals of the C group, from the twentieth day of administration until the end of experiments.

The production of cholesterol, the major constituent of the lipoproteins, may be enhanced by diabetes [36]. Our results showed that the cholesterol levels between the nondiabetic control group and treated group were statistically equal. An increase was observed in the level of cholesterol in the diabetic control. However, studies report that average values observed for the HDL cholesterol were significantly higher in diabetic rats. This could possibly provide protection against the development of atherosclerotic macrovascular disease in diabetic animals, contrary to what is observed in humans. The genesis of these findings has not yet been elucidated [22].

*A. humile* extract reduced the levels of total cholesterol and no significant difference was observed in either treated or control rats (Table 1). El-Missiry and El Gindy [15] and Benwahhoud et al. [37] obtained similar results using *Suaeda fruticosa* extract, while the administration of *Spergularia purpurea* aqueous extract was ineffective in reducing cholesterol levels [38]. Both cholesterol increase and reduction have been reported after alloxan administration in rats [38].

The administration of the *A. humile* extract decreased by 21% the rates of alanine aminotransferase (ALT) in the diabetic rats, when compared to control animals at the end of the treatment, suggesting a hepatic protection of *A. humile* against the damaging effects of diabetes (Table 1). Also, results showed that *A. humile* extract did not change the pancreatic insulin levels during the experimental period (Table 1), suggesting no side effects of this treatment.

Indeed, among the factors related to the effects of administration of *A. humile*, the decrease in insulin degradation by the inhibition of the enzyme insulinase [39] or the increase in the outlying sensibility of the existent insulin receivers in the *β*-cell [25] or even direct activation of these same receptors [40] may be considered. In addition, the hyperglycemia reduction observed in the present study could be related to a possible decrease of glucose absorption due to the inhibition of the *α*-amylase secretion responsible for the increase of both glucose and triglycerides in the metabolism [41]. Finally, the possibility that the bioactive metabolites act on modulating the synthesis and secretion of insulin should not be excluded [42].

## 4. Conclusions

In conclusion, the use of aqueous extracts and isolated natural products, exclusively derived from plants, in alternative therapies for antidiabetes drugs has been increasing. Obtained results demonstrate that the administration of *Anacardium humile* aqueous extract to alloxan-induced diabetic rats is effective in regulating the levels of blood glucose and other related parameters. However, it does not seem to alter insulin secretion into the blood stream and shows no hepatic or renal toxicity. Analysis of the different bioactive compounds of *A. humile* aqueous extract, as well as the preclinical and clinical toxicity evaluation followed by the determination of therapeutic intervals, should insure the efficacy and safety of *A. humile* in the treatment of diabetes in humans. The potential use of this extract or its isolated compounds as alternative treatment and/or as molecular models for the development of new therapeutic agents in the treatment of diabetes or other diseases needs to be evaluated in future studies.

## Figures and Tables

**Table 1:** Total cholesterol, insulin, and ALT levels on the 28th day of treatment.

Group	*N*	Treatment	Cholesterol (mg/dL)	Insulin (*µ*UI/mL)	ALT (UI/L)
C	6	Saline	53.83 ± 0.79^b^	0.0800 ± 0.0044^a^	64 ± 23.11^b^
D	6	Saline	70.66 ± 1.64^a^	0.0383 ± 0.0044^b^	165.3 ± 22.01^a^
T	6	*A. humile* (175 mg/kg)	59.33 ± 2.18^b^	0.0383 ± 0.0044^b^	146 ± 22.11^ab^

Mean values with different letters are significantly different at *P* < 0.05 (average ± SD)*.*

C: control

D: diabetic rats

T: treated rats.
